# Better safe than so ray: national survey of radiation protection amongst interventional radiology trainees in the United Kingdom

**DOI:** 10.1259/bjr.20230071

**Published:** 2023-07-25

**Authors:** S Patel, P Jenkins, J Zhong, W Liu, K Harborne, S Modi, C Joy, R Williams, P Haslam

**Affiliations:** 1 University Hospital Southampton, Southampton, United Kingdom; 2 UK National Interventional Radiology Trainee Research (UNITE) Collaborative, London, UK; 3 Peninsula Radiology Academy, Plymouth, United Kingdom; 4 Department of Diagnostic and Interventional Radiology, Leeds Teaching Hospitals NHS Trust, Leeds, UK; 5 University Hospitals Coventry and Warwickshire, Coventry, United Kingdom; 6 University Hospitals Birmingham NHS Foundation Trust, Birmingham, United Kingdom; 7 Royal Free Hospital, Royal Free London NHS Trust, London, United Kingdom; 8 Freeman Hospital, The Newcastle Upon Tyne Hospitals NHS Foundation, London, United Kingdom

## Abstract

**Objective:**

To establish the provision and use of radiation personal protective equipment (PPE) and dosimetry amongst UK interventional radiology (IR) trainees and highlight areas of improvement in order to enhance the radiation safety.

**Methods:**

A survey questionnaire was designed by members of the British Society of Interventional Radiology (BSIR) trainee committee via survey monkey and distributed to UK IR trainees via the BSIR membership mailing list, local representatives and Twitter. The survey was open from 04/01/2021 to 20/02/2021. Only IR trainees in years ST4 and above were included.

**Results:**

Of the 73 respondents, 62 qualified for analysis. Respondents (81% male) spent a median of 5.5 sessions (half day list) per week in the angiography suite and 58% (n=36) had difficulty finding appropriately sized lead aprons at least once a week. Overall 53% (n=33) had concerns about their radiation PPE. Furthermore 56% of trainees (n=35) experienced back pain among other symptoms attributed to wearing the lead aprons available to them. 77% (n=48) regularly wore lead glasses. For trainees requiring prescription glasses (n=22) overfit goggles were provided however 17 (77%) of these trainees felt the goggles compromised their ability to perform the procedure. Eye and finger dosimeters were used by 50% and 52% of respondents respectively. Compliance with body dosimetry was 99%.

**Conclusion:**

Provision of radiation PPE and dose monitoring for IR trainees is suboptimal, particularly access to adequate eye protection or suitably fitting leads. Based on the findings of this survey, recommendations have been made to promote the safety and radiation awareness of IR trainees.

**Advances in knowledge:**

Radiation protection practices for IR trainees nationally are poor. Provision of suitable eye protection and well fitting lead body protection is low.

## Introduction

Interventional radiology (IR) trainees in the United Kingdom (UK) complete three years of dedicated IR training with a recommended 75% of their time spent in direct clinical activity related to intervention.^1^ A large proportion of this time is spent performing procedures using fluoroscopy or computed tomography (CT), which involve radiation exposure to the operator. In the UK, such practice is subject to regulation and employers have duties under Ionising Radiations Regulations (IRR) 2017 to ensure their employees do not exceed any radiation dose limits to the body, and specifically lens of the eye.

There is a shortage of interventional radiologists within the UK in particular to ensure appropriately staffed out of hours, emergency care.^2^ Concerns about radiation safety feature highly within radiology training and are a key focus of the Fellowship of the Royal College of Radiologists (FRCR) Part A Physics exam with the adoption of the linear no threshold theory and how it relates to the UK regulatory framework forming part of the syllabus.^3^ At low doses, the stochastic effects including the risk of malignancy remain concerning and poorly understood, A recent systematic review of the available evidence suggests a higher preponderance of left-sided brain tumours and cataract development.^4^ Radiation protection is a legal obligation, and it is essential for training schemes to address any radiation concerns and ensure IR trainees are appropriately protected.

We aim to establish the provision and use of radiation personal protective equipment (PPE) and dosimetry amongst IR trainees (ST4-6) and fellows within the UK to highlight areas of improvement in order to enhance radiation safety in IR.

## Material and methods

This was a national cross-sectional study using a questionnaire survey designed by members of the British Society of Interventional Radiology (BSIR) trainee committee and UK National Interventional Radiology Trainee Research (UNITE) Collaborative^5^ and approved by the BSIR Audit and Registry committee. The domains the questionnaire was designed to capture included time spent in the interventional angiography suite, provision of PPE, compliance with wearing PPE, PPE comfort, adverse symptoms related to PPE, and dosimetry use.

Data were collected in accordance with general data protection regulations. The survey (see appendix) was created in Survey Monkey^®^ and distributed to UK IR and interventional neuroradiology (INR) trainees via the BSIR membership mailing list, local representatives and the Twitter social media platform. The survey was open from 04/01/2021 to 20/02/2021.

Only IR/ INR trainees were considered for the analysis, defined as those in IR/ INR subspecialty training year ST4 and higher or in a dedicated post certificate of completion of training (post-CCT) IR fellowship programme as testified by themselves. Diagnostic Radiology trainees and ST1-ST3 trainees were excluded from the study. All descriptive statistics were performed on Microsoft Excel (Microsoft Corporation, 2018). Logistic regression was performed using StataCorp (College Station, TX: StataCorp LLC, version 17) to find associations between trainee demographics (age, gender, height, weight), number of IR sessions and PPE use (how often trainees had difficulty finding suitable fitting lead aprons) with their self-reported incidence of back pain. Ethical committee approval was not required.

## Results

### Demographics

Over the study period, a total of 73 responses were received. Eleven respondents were not in a registered IR training programme/fellowship status and were excluded from the analysis. Of the remaining 62 respondents, 27% (*n* = 17) were ST4, 48% (*n* = 30) were ST5, 20% (*n* = 12) were ST6 and 5% (*n* = 3) were post-CCT IR fellows. Male trainees made up 81% (*n* = 50) of respondents. The median age was 33 years (range 28–43). The median number of sessions per week (defined as half day list) in the intervention suite was 5.5 (range 1–10). In total, 25 deaneries were represented. Overall 53% (*n* = 33) had concerns about their radiation PPE.

### Lead aprons and thyroid shields

With regard to PPE, only 5% (*n* = 3) had their own personal lead apron. These were all purchased by the individual trusts the trainee worked in. Only 10% (*n* = 6) had their own personal thyroid shield. Of these individuals, four had been paid for by the individual trust and two by industry.

**Figure 1.**
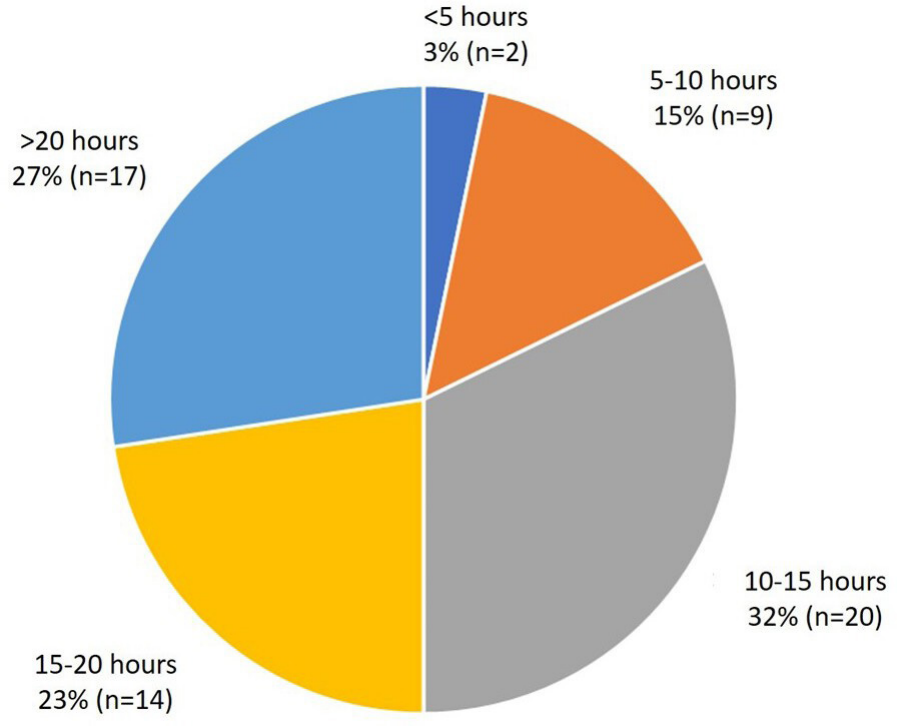
Pie chart showing number of hours of week spent wearing lead by respondents

**Figure 2.**
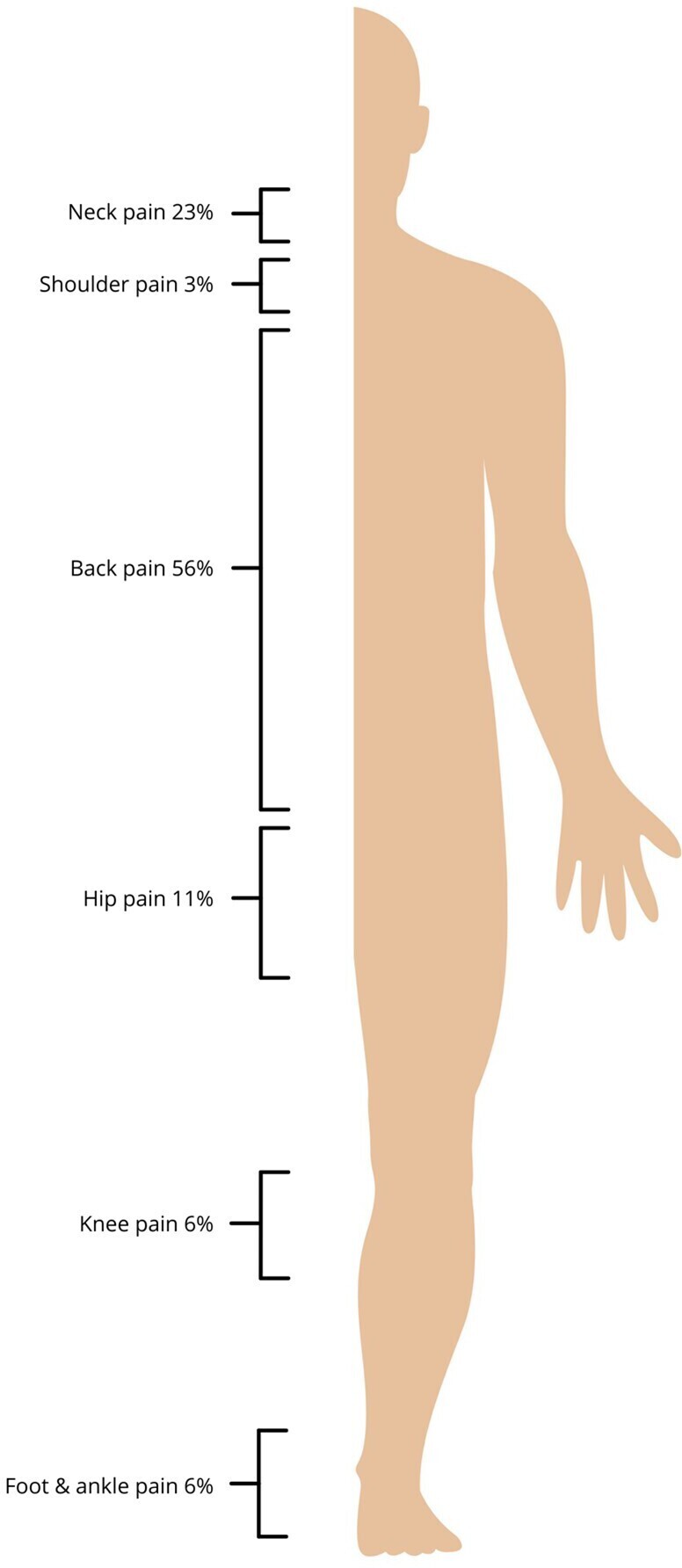
Diagram showing percentage of respondents reporting adverse symptoms as result of the lead aprons provided locally

The number of hours each week spent wearing by the respondents is shown in Figure 1, with 82% (*n* = 51) wearing lead aprons for over 10 h per week. When asked about adverse symptoms felt to be related to the wearing of lead aprons, 56% (*n* = 35) experienced back pain, 23% (*n* = 14) experienced neck pain, 11% (*n* = 7) had hip pain, 6% (*n* = 4) had knee pain, 6% (*n* = 4) had foot or ankle pain and 3% (*n* = 2) felt shoulder pain was an issue (Figures 2).

**Figure 3.**
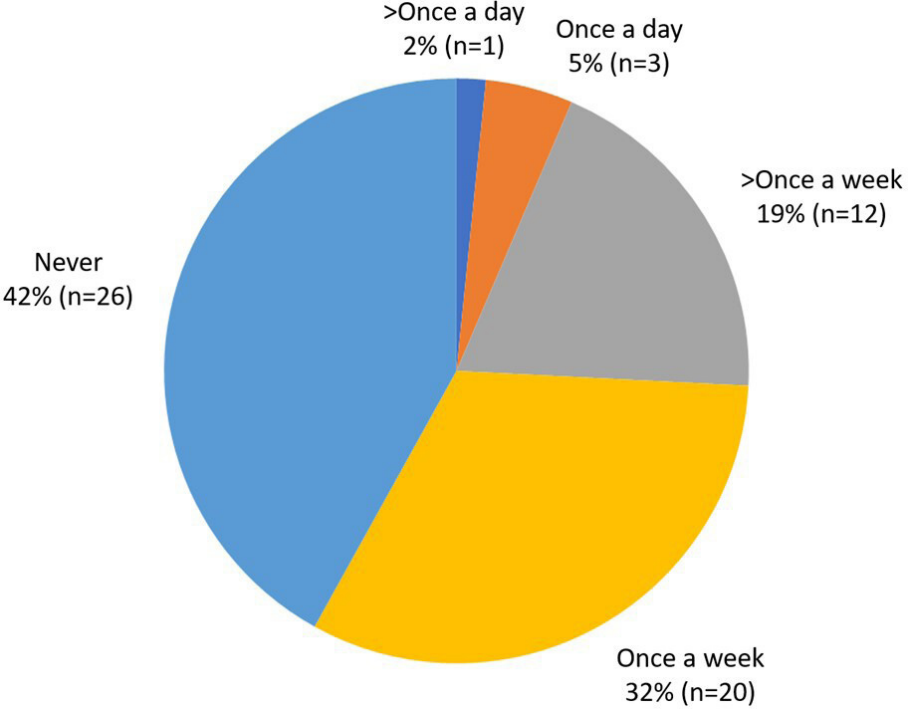
Pie chart showing how often respondents had difficulty findings appropriately fitting lead aprons.

When asked how frequently trainees had difficulty finding appropriately fitting lead aprons, 51% (*n* = 32) stated that they did once a week or more and 6% (*n* = 4) had difficulty once a day or more (Figure 3).

Increased frequency of wearing ill-fitting lead aprons was associated with back pain (*p* = 0.03, OR 2.13, 95% CI 1.09–4.14), whereas age, gender, height, weight and number of IR sessions were not associated (*p* < 0.05).

### Eyewear

Lead glasses were available for the majority of individuals, with 48/62 (77%) using eye protection on a regular basis, with 42% (26/62) wearing personal lead glasses and of these, 70% (18/26) were purchased by the individual trust, 4% (2/26) by the training scheme, 19% (5/26) were self-funded and one individual shared the cost with the trust. 31% (8/26) required prescription lenses which were funded by the individual trusts.

For those trainees not wearing personal radiation protection glasses, all 22 said that they were provided with overfit goggles. When asked if they felt the overfit goggles compromised their ability to perform a procedure, 77% (*n* = 17) answered “yes”.

### Dosimetry

Trainees were provided with a list of dosimetry devices and asked to select which dosimeters they used; 99% (*n* = 61) use a body dosimeter under their lead apron, 50% (*n* = 31) used an eye dosimeter, 52% (*n* = 32) used finger dosimeters, 8% (*n* = 5) used collar/thyroid dosimeters and 5% (*n* = 3) used ankle dosimeters. Three respondents used real-time dosimetry devices.

## Discussion

This survey highlighted that IR trainees in the UK spend a significant portion of their working week wearing PPE; however, over half the respondents experienced difficulty finding appropriately fitting lead aprons and over half of the respondents experienced back pain that they attributed to their PPE. The logistic regression model to identify factors associated with this reported back pain also found that increased frequency of not finding appropriately fitting lead aprons during the week was associated with the reporting of back pain. Worryingly, over half the respondents had concerns about their radiation PPE. Additional concerns raised by this survey include the lack of suitably fitting eye protection, with 27% raising the concern that it compromised their performance along with inconsistent eye dosimetry provision. Very few trainees had funded personal PPE provided. This is despite IR trainees participating in a median of 5.5 sessions a week, equating to almost three full working days with 82% of respondents wearing lead aprons for a total estimated duration of over 10 h per week. Whether these individuals are receiving or have the potential to receive doses in excess of the classification thresholds according to the Health and Safety Executive^6^ requires further investigation but this survey highlights the variation in dosimetry provision and use amongst UK IR trainees. Under regulation 9 of Working with ionising radiation : Ionising Radiations Regulations 2017^6^ the employer has a duty “to take all necessary steps to restrict so far as reasonably practicable the extent to which its employees and other persons are exposed to ionising radiation”. Employers must designate employees as classified if they are “likely to receive an effective dose greater than six millisieverts (mSv) per year or an equivalent dose greater than 15 mSv per year for the lens of the eye or greater than 150 mSv per year for the skin or the extremities”.^6^ Although not all IR team members, including IR trainees, are likely to exceed these classification thresholds, the decision to classify a worker must be informed by risk assessment.

All employees have a duty under section 7 of the Health and Safety at Work Act (HSWA) to cooperate with the employer so far as is necessary so that the employer may comply with their duties.^7^ For employees working with ionising radiation, they must comply with the employer’s dosimetry requirements and instruction to wear the appropriate radiation PPE. Health and Safety Executive (HSE) guidance, highlighted in the PPE at Work Regulations 1992 states that “If PPE is still needed after implementing other controls, you [the employer] must provide this for your employees free of charge” and to “Choose equipment that suits the user – consider the size, fit and weight of the PPE. If the users help choose it, they will be more likely to use it”.^8^

Scattered dose from the patient presents the most significant dose to the interventional radiologist and staff within the angiography suite.^9,10^ In 2017, the eye lens dose limit was reduced to 20 mSv per year, from 150 mSv per year in the.^6^ This has been reduced due to increased understanding of the deterministic effect of radiation on the eye lens causing cataracts.^11,12^ This change led to the increased availability and use of lead glasses, and likely results in the increased awareness amongst trainees of this regulation. Provision of eye protection was present in the majority of cases, however almost a quarter of IR trainees were still not using eye protection on a regular basis which could be partly attributed to under provision of suitable fitting eye protection given that less than half of trainees were provided with their own pair.

The provision of appropriate eye protection is mandated by the Ionising Radiations Regulations 2017^6^ and is the responsibility of the employer. However, nearly a quarter of those trainees wearing personal eye protection had still been required to fund or part fund the acquisition of lead goggles at significant cost of around £300-£500. Of further concern is the arrangement of overfit goggles, lead glasses that are designed to fit over existing non-lead eyewear. These are not infrequently encountered within the radiology department and represent a much cheaper option due to the reduced cost of manufacturing and the shared use between multiple users with different ocular prescriptions. 77% of those who had been required to use these stated that they felt this compromised their ability to perform the procedure. This is particularly concerning from a trainee perspective where there is already pressure to maximise their finite training opportunities with significant disruptions recently from the COVID-19 pandemic and reduction in areas of elective IR work.^13,14^ One additional concern is for patient safety if the trainee operator’s vision is impaired due to ill-fitting goggles. Depending on the design, radiation protection glasses have been shown to significantly attenuate scattered dose to the lens.^15^ An urgent requirement for individual leads goggles to be provided at the time of appointment to an IR training post (usually at ST4) by the employer is recommended.

Published case series have also highlighted interventional radiologists and cardiologists may be at increased risk of head and neck neoplasms. Disproportionate reports of left-sided brain tumours, the side-commonly receiving higher radiation doses, raises the possibility of a causal relation to occupational radiation exposure.^4,16^ What the survey does not capture is the availability of additional radiation protection options within the IR suite such as the use of lead screens and protective drapes however.

The requirement for employer provision of PPE is complicated in the UK training system because both individual trusts and Health Education England (HEE) (as the responsible training body) are responsible in part for the full employment of trainees. Similarly, as trainees move around trusts on a regular (usually 6 monthly or yearly basis), they typically become employees of different individual trusts over the course of their training. This raises funding questions, with some trusts reluctant to pay for prescription glasses if trainees are moving on soon and some view the responsibility lying with the training programme or deanery to provide adequate protection. However, the finding that only 31% of trainees received funding from either the trust or training scheme towards the cost and only 5% had their own personal lead apron remains a concern. With these findings coming to light, it is vital for local training schemes to work with individual NHS trusts and HEE to provide the appropriate PPE for trainees to ensure their work, training and health are not compromised.

Every IR department in the UK will generally have a range of lead aprons and thyroid shields for non-permanent staff or visitors that trainees who do not have their own personal lead aprons will use. Wearing heavy and uncomfortable ill-fitting lead aprons is associated with increased back pain amongst interventional laboratory staff, which can be work limiting.^17^ Furthermore, the number of years worked is associated with an increase in the incidence of spinal problems.^18,19^ In this relatively young cohort of IR trainees, we found that 56% already experienced back pain a result of the lead aprons they were provided with this was associated with the increased frequency of wearing poorly fitting lead aprons on a weekly basis. This highlights the urgent need improve the provision of suitably fitting lead for trainees, who are the future IR workforce in an already depleted subspecialty group where the IR consultant shortage is estimated to be at 28% with half of UK hospital trusts and health boards unable to provide 24/7 IR services.^20^ Although this survey cannot prove a direct relationship between ill-fitting leads and musculoskeletal issues, the survey question specifically asks about any symptoms felt to be caused by the lead apron therefore reflecting the respondents opinion. The lack of personal radiation PPE provision demonstrated within this survey of UK trainees is greatly concerning as this could impact on career choices and career longevity. Back pain is a leading cause of disability globally, a common cause of occupational-related sickness and associated with a high socioeconomic burden^21,22^ and with an average age of 33, most trainees will still have over 30 years left of their career, to consider the impact of fitting leads and improper radiation protection. Further measures to improve ergonomics within IR are also required.

Compliance with wearing body dosimetry was satisfactory with only one respondent not selecting this option from our survey; however, only 52% used an eye dosimetry device which may be due to variation in provision of eye dosimetry. Inconsistencies in eye dosimeter provision are likely due to different local departmental protocols; however, these results should highlight the need to standardise such monitoring protocols to ensure all IR trainees are adequately provided. No data are available on departmental provision of dosimeters in the respondent’s local trusts and would be important for future work. With the highlighted radiation risks to the lens, at a minimum employers should carry out risk assessments for IR trainees with advice from the local radiation protection advisor to determine the most appropriate level of ongoing monitoring required. Ultimately, the employer may be liable for prosecution if a dose limit was exceeded and, depending on the circumstances, the employee too.

Of additional interest was the finding that the majority of respondents (81%) were male, which reflects the current strong male preponderance of UK IR trainees and additional efforts and initiatives from the BSIR are required to address this. Concerns related to radiation risks in pregnancy were featured highly in a recent survey as reasons why a career in IR was less attractive to females.^23^ Radiation safety measures for the female interventionalist are paramount, given the additional considerations required during pregnancy and concerns over exposure to ionising radiation must be clearly addressed so that talented female trainees are not deterred by this.

### Limitations

This was an optional anonymous survey which was open to a wide number of respondents with no ability to verify whether the information provided was accurate or a fair reflection of their trusts. Naturally those who were concerned about radiation protection were more likely to respond, providing a respondent bias which cannot be mitigated. The estimated response rate was reasonable, based on the projected number of UK IR trainees to be between 100– and 20, although this number is challenging to estimate due to variable definitions and working practices, in particular relating to non-vascular intervention. This was still felt to be a fair representation of the status of radiation protection for IR trainees within the UK. Due to the nature of the survey, no dose data including classification status were reviewed.

#### Recommendations

- All IR trainees should be provided with their own lead goggles at commencement of their IR training post at ST4 by the local training scheme or employing trust.
- An assessment of lead apron and thyroid shield availability should be performed at induction within each employer trust with a dedicated set of leads assigned for a trainee. If no suitable well-fitting lead is found then a new personalised lead should be purchased by the training scheme or trust.
- Standardised national radiation monitoring protocols are required to reduce the variation in dosimeter provision.
- Local IR trainees should be re-evaluated regularly for dosimeter monitoring of their body, finger and eye doses, and designated as classified workers if required.

### Highlights

- Only 5% of UK IR trainees have personal lead aprons.
- 56% experienced back pain felt to be related to lead aprons.
- 58% had difficulty finding appropriately fitting lead aprons.
- 58% did not have their own pair of lead goggles and only 50% use eye dosimeters.
- 77% of trainees using overfit glasses report their performance is compromised.

## Conclusion

Provision of radiation PPE and dose monitoring of fingers and eyes for IR trainees are suboptimal, particularly access to adequate eye protection or suitably fitting leads. Radiation protection is an integral part of IR and trainees are subject to stringent requirements under.^6^ Provision of PPE is the employer’s responsibility. Recommendations based on the study findings have been made for the safety and benefit of IR trainees.
